# Efficacy of polyarginine peptides in the treatment of stroke: A systematic review and meta‐analysis

**DOI:** 10.1002/brb3.2858

**Published:** 2022-12-21

**Authors:** Fateme Tahmasbi, Arian Madani Neishaboori, Mahta Mardani, Amirmohammad Toloui, Khalil Komlakh, Yaser Azizi, Mahmoud Yousefifard

**Affiliations:** ^1^ Student Research Committee Tabriz University of Medical Sciences Tabriz Iran; ^2^ Physiology Research Center Iran University of Medical Sciences Tehran Iran; ^3^ School of Medicine Tehran University of Medical Sciences Tehran Iran; ^4^ Department of Neurosurgery, Imam Hossein Hospital Shahid Beheshti University of Medical Sciences Tehran Iran; ^5^ Department of Physiology, School of Medicine Iran University of Medical Sciences Tehran Iran

**Keywords:** brain edema, functional status, infarction, polyarginine, Stroke

## Abstract

**Background:**

Disparities exist regarding an efficient treatment for stroke. Polyarginines have shown promising neuroprotective properties based on available published studies. Thus, the present study aims to systemically review and analyze existing evidence regarding polyarginine's administration efficacy in animal stroke models.

**Method:**

Medline, Scopus, Embase, and Web of Science were systematically searched, in addition to manual search. Inclusion criteria were administrating polyarginine peptides in stroke animal models. Exclusion criteria were previous polyarginine administration, lacking a control group, review articles, and case reports. Data were collected and analyzed using STATA 17.0; a pooled standardized mean difference (SMD) with a 95% confidence interval (CI), meta‐regression, and subgroup analyses were presented. Risk of bias, publication bias, and level of evidence were assessed using SYRCLE's tool, Egger's analysis, and Grading of Recommendations Assessment, Development and Evaluation framework, respectively.

**Results:**

From the 468 searched articles, 11 articles were included. Analyses showed that R18 significantly decreases infarct size (SMD = –0.65; 95% CI: –1.01, –0.29) and brain edema (SMD = –1.90; 95% CI: –3.28, –0.51) and improves neurological outcome (SMD = 0.67; 95% CI: 0.44, 0.91) and functional status (SMD = 0.55; 95% CI: 0.26, 0.85) in stroke animal models. Moreover, R18D significantly decreases infarct size (SMD = –0.75; 95% CI: –1.17, –0.33) and improves neurological outcome (SMD = 0.46; 95% CI: 0.06, 0.86) and functional status (SMD = 0.35; 95% CI: 0.16, 0.54) in stroke models.

**Conclusion:**

Moderate level of evidence demonstrated that both R18 and R18D administration can significantly improve stroke outcomes in animal stroke models. However, considering the limitations, further pre‐clinical and clinical studies are warranted to substantiate the neuroprotective efficacy of polyarginines for stroke.

## Graphical abstract

The findings are summarized in the above figure. Both R18 and R18D showed promising effects on decreasing the infarct size, enhancing functional status, and improving neurological outcome in animal models of stroke. In addition, R18 administration was effective in decreasing brain edema as well.

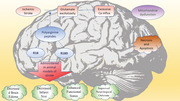

## 1 INTRODUCTION

Stroke is a medical condition defined as a neurological deficit caused by an acute vascular injury in the central nervous system (CNS). Stroke could be attributed to cerebral infarction, intracerebral hemorrhage (ICH), and subarachnoid hemorrhage (SAH) (Sacco et al., 2013). Stroke is one of the leading causes of mortality and morbidity worldwide. Although the prevalence of stroke has decreased in recent years; the age, sex, and geographic variance of the patients have increased, leading to the increased socioeconomic burden of stroke according to the Global Burden of Disease studies (Beghi et al., 2019; Katan & Luft, 2018).

The primary goal in managing stroke is to restore the blood flow of the affected region as fast as possible (Kuriakose & Xiao, 2020). Endovascular thrombectomy, based on injecting tissue plasminogen activator (tPA), is mainly used to resolve vascular occlusion in patients with stroke. However, tPA administration is limited by many factors for instance, it can only be administered in the first 4.5 h from the onset of stroke. Furthermore, it is contraindicated in many patients, including patients with a history of head trauma, stroke, SAH, intracranial neoplasm, elevated blood pressure (Hughes et al., 2018). Therefore, finding new approaches to control and minimize the adverse effects of stroke on patients' lives has always been a research priority.

Cationic arginine‐rich cell‐penetrating peptides (CARPs) have been addressed by scholars for their neuroprotective effects on CNS (Meloni et al., 2017a). CARPs are short peptides, usually consisting of 5–25 amino acids, facilitating the delivery of many macromolecules and molecules, including other peptides, proteins, nucleic acids, and drugs to cells. The most widely used CARPs are polyarginine peptides, PEP‐1 peptides, trans‐activator of transcription (Tat) protein, and transportin. Existing evidence has indicated that this neuroprotection is achieved by reducing neuronal calcium flow and, therefore, minimizing neurotoxicity (Meloni, Milani, et al., 2015).

In addition, polyarginine peptides and other arginine‐rich peptides like TAT are hypothesized to have natural neuroprotective features, and these neuroprotective properties seem to increase with increasing the arginine content. Laboratory evidence has also shown that polyarginine treatment reduces the volume of the infarct area following continuous occlusion of the middle cerebral artery (MCAO) in rats (Meloni, Brookes, et al., 2015). Peptides containing polyarginine seven have had neuroprotective effects in in vitro studies in a model of glutamate‐induced excitatory toxicity (Marshall et al., 2015; Meloni, Brookes, et al., 2015).

Previous in vivo and in vitro studies revealed that polyarginine peptides could lead to improved stroke outcomes in various aspects. Therefore, this systematic review and meta‐analysis aimed to investigate the role of polyarginines in animal stroke models and expand the knowledge on the role of polyarginine peptides in the current literature.

## 2 METHOD

This systematic review was performed according to the Preferred Reporting Items for Systematic Reviews and Meta‐Analyses statement (PRISMA) recommendations (Moher et al., 2009).

### 2.1 Information sources and search strategy

The PICO framework was applied to develop the search strategies. Accordingly, population (P) was animal models of cerebral ischemia, intervention (I) was administrating polyarginine peptides, control (C) was not receiving polyarginine peptides, and outcome (O) was the extent of the cerebral defect in terms of cerebral edema and infarct size, sensory‐motor function assessment (neurological outcome assessment) and motor function assessment based on motor evaluation tests.

Several medical databases, including Medline (using PubMed), Embase, Scopus, and Web of Science, were comprehensively searched for relevant articles from inception to March 2021. Google Scholar and the reference lists of the selected articles and related review articles were also screened to ensure complete literature inclusion. Studies that fit the inclusion criteria were imported as well. The full search syntaxes for the four databases are presented in Appendix 1.

### 2.2 Eligibility criteria and study selection

Studies were included in cases of administering intracranial, intraperitoneal, or intravenous polyarginine peptides for cerebral ischemia/reperfusion animal models. The exclusion criteria were as follows: using pretreatment by polyarginine peptides, lacking a control group with ischemia, in vitro studies, not reporting the interested data, non‐rodent studies, review articles, case reports, and retracted papers. After removing the duplicates, two independent reviewers performed the screening process.

The study selection process was conducted by screening the titles and abstracts of the imported studies, and irrelevant articles were removed. The full texts of the remaining studies were thoroughly screened. Ten articles were excluded, five of them due to unavailability of full‐text, three due to irrelevant subjects, and two were review articles.

### 2.3 Data collection process and data items

Two authors independently extracted the data from the included studies to a predefined Excel sheet. The following data were extracted from each of the included studies: bibliographic data (name of the first author, year and origin of the publication), methodology data (number, species, stroke model, age, duration between the induction of stroke and treatment, follow‐up time interval type, dosage, and the administration route of the polyarginine peptide), and main outcomes.

### 2.4 Risk of bias and quality assessment

The risk of bias of the included studies was assessed using the SYRCLE tool (Hooijmans et al., 2014). This tool is an adaptive version of the Cochrane risk of bias tool. It contains 10 entries related to selection bias, performance bias, detection bias, attrition bias, reporting bias, and other biases.

### 2.5 Statistical analysis

The analysis of data was conducted by STATA 17.0 software. The outcomes were categorized into neurological outcome, functional status, infarct size, and cerebral edema. The effect size was estimated for different polyarginines on each of the outcomes by calculating standardized mean difference (SMD). Then, based on the Hartung–Knapp–Sidik–Jonkman modification, a 95% confidence interval (95% CI) was calculated (IntHout et al., 2014). Afterward, *I* ^2^ statistic was applied to estimate heterogeneity in the studies (Higgins & Thompson, 2002). *I* ^2^ > 50% or *p*‐value of less than 0.1 was considered as the presence of heterogeneity. In cases with no heterogeneity, a fixed‐effect model, and otherwise, a random‐effect model was applied. In cases of a sufficient number of trials, subgroup analysis was conducted for the stroke outcomes and the administered polyarginine. Then, using meta‐regression, a coefficient (Coef.) and *p*‐value were presented for each of the subgroups to determine the source of the heterogeneity. Based on the meta‐regression, a *p*‐value of less than .05 was considered as a source of heterogeneity. Furthermore, Egger's test was applied to estimate the publication bias (Egger et al., 1997).

### 2.6 Certainty of evidence

The level of evidence was evaluated using the Grading of Recommendations Assessment, Development and Evaluation (GRADE) framework for animal studies (Wei et al., 2016).

## 3 RESULTS

### 3.1 Study selection

**FIGURE 1:**
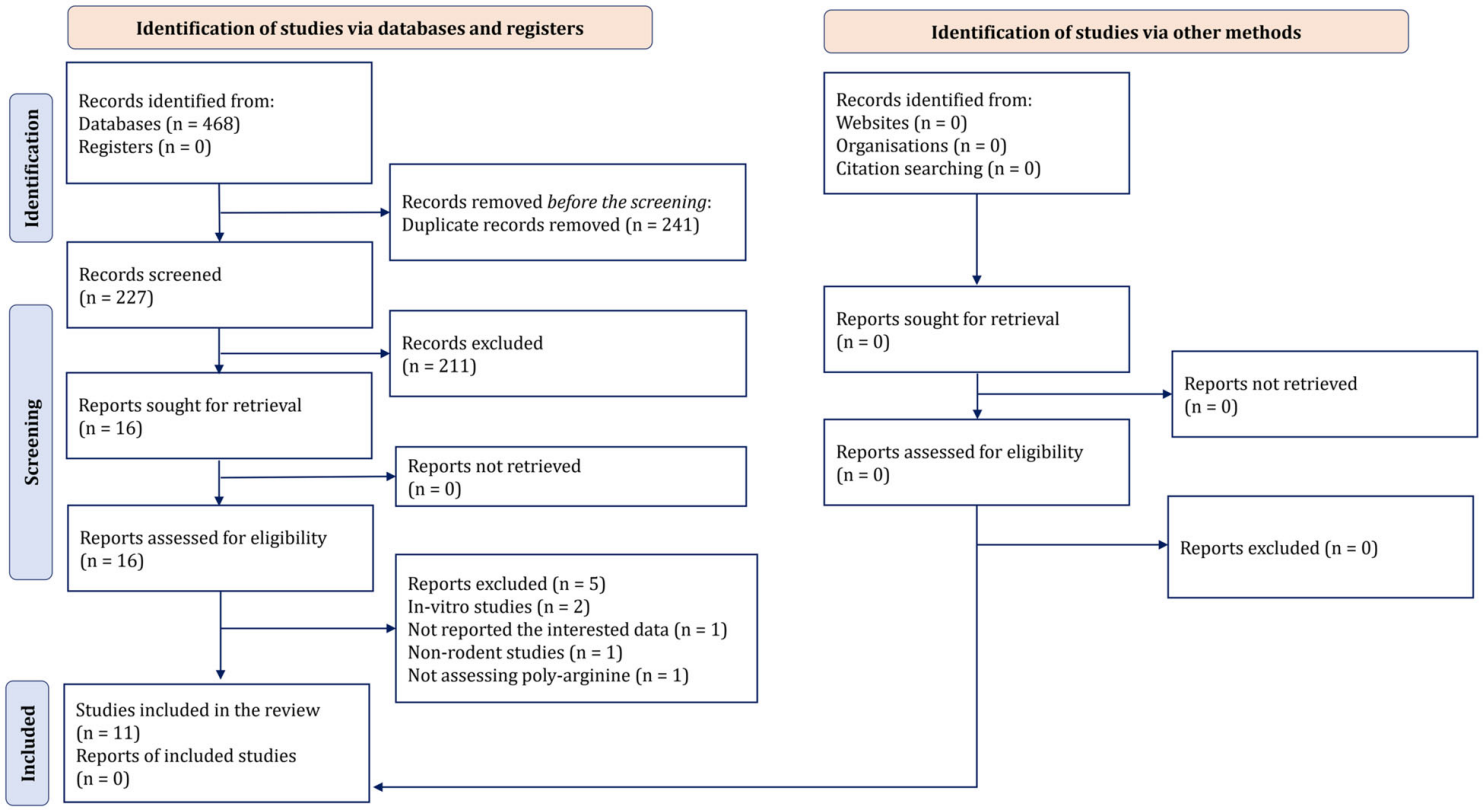
Prisma flow diagram of the present study. Of the 227 unduplicated searched articles, 11 articles were entered to the present study.

Figure 1 demonstrates the selection of studies throughout the search process. The systematic and manual search generated 475 records. After the deduplication, 227 articles remained to be subjected to inclusion and exclusion criteria. With inclusion and exclusion criteria imposed first on the titles and abstracts and second on study design and methods, only 11 citations entered this systematic review (Edwards et al., 2018a; Edwards, Cross, et al., 2018; Hyun et al., 2010; Meloni, Brookes, et al., 2015; Meloni et al., 2017b; Meloni et al., 2019; Milani et al., 2018, 2021, 2017; Milani, Clark, et al., 2016; Milani, Knuckey, et al., 2016).

### 3.2 Characteristic of the included studies

The studies were conducted from 2010 to 2021; 10 studies were conducted on Sprague–Dawley rats (Edwards et al., 2018a; Edwards, Cross, et al., 2018; Hyun et al., 2010; Meloni, Brookes, et al., 2015; Meloni et al., 2017b; Meloni et al., 2019; Milani, Clark, et al., 2016; Milani et al., 2021, 2017; Milani, Knuckey, et al., 2016) and one on Wistar rats (Milani et al., 2018). Regarding the stroke model, nine studies implemented the MCAO model, and two studies the Rice–Vannucci HI model (Edwards et al., 2018a; Edwards, Cross, et al., 2018). The duration of stroke varied among studies, ranging from 10–20 min to 180 min. Furthermore, different types of polyarginine peptides were administered: R18, R18D, RD9, R15, and R12. However, analysis was performed on R18 and R18D, merely due to the small number of studies regarding other polyarginines. Analysis was performed concerning the priorly mentioned stroke outcomes of infarct size, neurological outcome, and functional status. Data regarding brain edema was not adequate for subgroup analysis. The means of administration were intravenously (IV) in eight studies, intraperitoneally (IP) in two studies, and intracranially (IC) in one study. Additional data are provided in Table 1.

**TABLE 1:** characteristics of the included studies

Author; year; country	Gender; strain; species	Stroke model; duration of arterial occlusion	Type of the administered polyarginine	Administered doses	Administration route	Interval time between stroke and treatment (minute)	Follow‐up time (hour)	Assessed outcomes	Sample size (stroke + treated)
Edwards; 2018; Australia (Edwards et al., 2018a)	NR; SD; rat	Rice‐Vannucci HI model; permanent	R18D	10, 30, 100, 300, 1000	IP	30, 60, 120	48	Infarct size; neurological outcome; functional outcome	166
Edwards; 2018; Australia (Edwards, Cross, et al., 2018)	NR; SD; rat	Rice‐Vannucci HI model; permanent	R18D, R18	30, 100, 300, 1000	IP	30, 60, 120	48	Infarct size; neurological outcome; functional outcome	106
Hyun; 2010; South Korea (Hyun et al., 2010)	Male; SD; rat	MCAO; 60 min	RD9	1000	IC	0	24	Infarct size	30
Meloni; 2015; Australia (Meloni, Brookes, et al., 2015)	Male; SD; rat	MCAO; permanent	RD9	1000	IV	30	24	Infarct size	18
Meloni; 2017; Australia (Meloni et al., 2017b)	Male; SD; rat	MCAO; permanent	R18	30	IV	30	24	Infarct size; neurological outcome; cerebral edema; functional outcome	20
Meloni; 2019; Australia (Meloni et al., 2019)	Male; SD; rat	ET‐1 MCAO; 10–20 min	R18D, R18	100, 300, 1000	IV	60	1344	Functional outcome	81
Milani; 2016; Australia (Milani, Clark, et al., 2016)	Male; SD; rat	MCAO; permanent	R12, R15, R18	1000	IV	60	24	Infarct size; neurological outcome; functional outcome	37
Milani; 2016; Australia (Milani, Knuckey, et al., 2016)	Male; SD; rat	MCAO; permanent	R18	100, 300, 1000	IV	60	24	Infarct size; neurological outcome; functional outcome	42
Milani; 2017; Australia (Milani et al., 2017)	Male; SD; rat	MCAO; permanent	R18	1000	IV	120	24	Infarct size; neurological outcome; functional outcome; cerebral edema	16
		MCAO; 180 min	R18	100	IV	120	24		14
		MCAO; 120 min	R18	1000	IA	120	24		17–18
Milani; 2018; Australia (Milani et al., 2018)	Male; Wistar; rat	MCAO; permanent	R18, R18D	300	IV	30	24	Infarct size; neurological outcome; functional outcome; cerebral edema	29
Milani; 2021; Australia (Milani et al., 2021)	Male; SD; rat	MCAO; Permanent (rats were euthanized after 4 h)	R18	300, 1000	IV	10	4	Infarct size	17
			R18D	300	IV	10	4		12

Abbreviations: IC, intracranial; IP, intraperitoneal; IV, intravenous; MCAO, Middle Cerebral Artery Occlusion; NR, not reported; SD, Sprague–Dawley.

### 3.3 Results of quality assessment and publication bias evaluation

**FIGURE 2:**
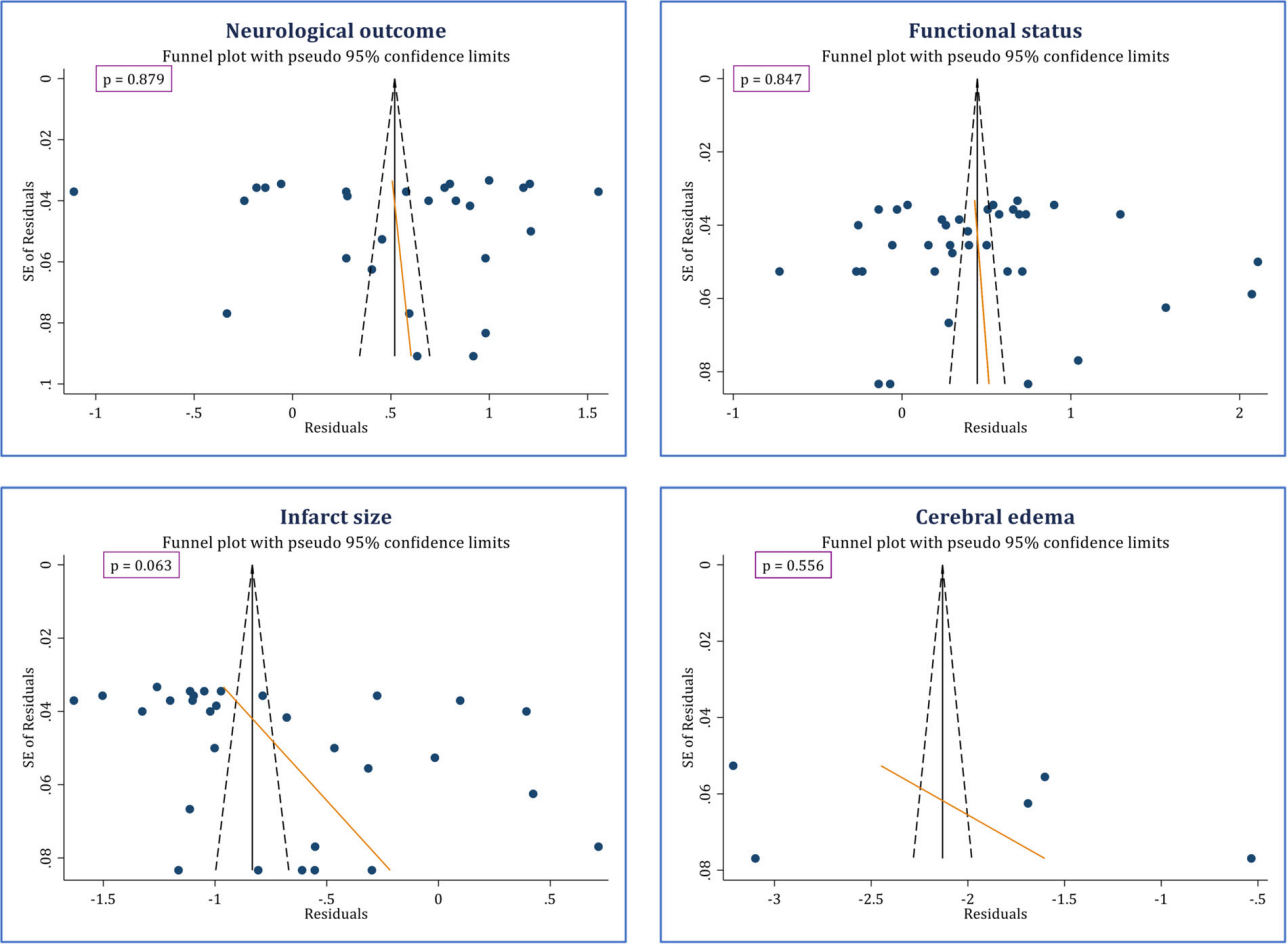
Publication bias evaluation of the present study. The funnel plots confirm no publication bias regarding the outcomes of the present study.

Table 2 shows the details on the quality assessment of included studies using SYCLE's tool. Accordingly, all studies had an unclear risk of bias for selective outcome reporting, random outcome assessment, random housing, and allocation concealment. Moreover, all studies had a low risk of bias as far as baseline characteristics were considered. When considering items of sequence generation, blinding trial caregivers, incomplete outcome data, and other sources of bias, all studies had a low risk of bias, except for one study, which was rated as having an unclear risk of bias in all of the three items (Hyun et al., 2010). In addition, no evidence of publication bias was observed concerning the effects of polyarginines on any of the outcomes of the present study (Figure 2).

**TABLE 2:** SYRCLE's risk of bias tool for the included studies

Study	Item 1: Sequence generation	Item 2: Baseline characteristics	Item 3: Allocation concealment	Item 4: Random housing	Item 5: Blinding trial caregivers	Item 6: Random outcome assessment	Item 7: Blinding outcome assessors	Item 8: Incomplete outcome data	Item 9: Selective outcome reporting	Item 10: Other sources of bias
Edwards et al. (2018)	L	L	U	U	L	U	L	L	U	L
Edwards et al. (2018b)	L	L	U	U	L	U	L	L	U	L
Hyun et al. (2010)	U	L	U	U	U	U	U	U	U	U
Meloni Brooks et al. (2015)	L	L	U	U	L	U	U	L	U	L
Meloni et al. (2017b)	L	L	U	U	L	U	U	L	U	L
Meloni et al. (2019)	L	L	U	U	L	U	U	L	U	L
Milani, Clark, et al. (2016)	L	L	U	U	L	U	L	L	U	L
Milani, Knuckey, et al. (2016)	L	L	U	U	L	U	L	L	U	L
Milani et al. (2017)	L	L	U	U	L	U	L	L	U	L
Milani et al. (2018)	L	L	U	U	L	U	L	L	U	L
Milani et al. (2021)	L	L	U	U	L	U	L	L	U	L

Abbreviations: H, high risk; L, low risk; U, unclear.

### 3.4 Effects of polyarginine administration on neurological outcomes

**FIGURE 3:**
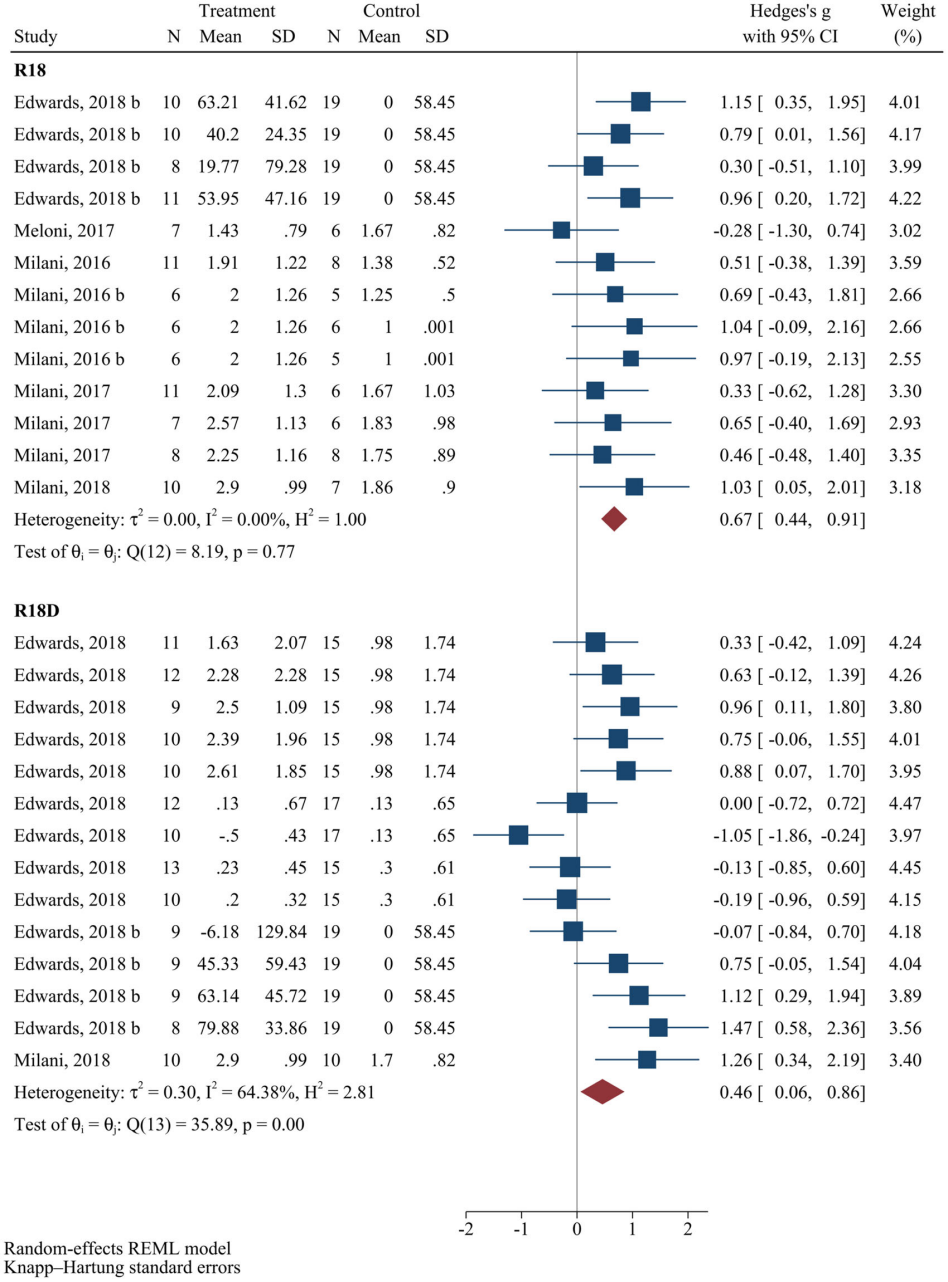
Efficacy of polyarginines’ administration on neurological improvement following ischemic stroke. Results demonstrated that R18 and R18D both significantly improved neurological outcomes following ischemic stroke.

Figure 3 shows the meta‐analysis results regarding the neurological outcomes after administrating R18 and R18D. Our results showed a significant improvement in neurological status following administration of R18 (SMD = 0.67; 95% CI: 0.44–0.91; *p* < .0001) and R18D (SMD = 0.46; 95% CI: 0.06–0.86; *p* = .027) in animal models of stroke. Worth mentioning that moderate heterogeneity was observed concerning the efficacy of R18D administration (*I* ^2^ = 64.38%, *p* < .001) in resolving neurological outcomes, while no heterogeneity was observed concerning that of R18 administration (*I* ^2^ = 0.00%, *p* = .77). Subgroup analysis was performed with regard to the administration of R18 and R18D.

Regarding R18, the results of subgroup analysis revealed that in terms of the time interval between injury to treatment, insignificant improvement in neurological outcome was only observed when R18 was administered 30 min after the injury (SMD = 0.38; 95% CI: −7.94 to 8.71; *p* = .661), compared to its administration immediately (SMD = 0.80; 95% CI: 0.23–1.37; *p* = .021), 60 min (SMD = 0.76; 95% CI: 0.35–1.16; *p* = .010) and 120 min (SMD = −0.46; 95% CI: 0.07–0.85; *p* = .035) (Table 3). Assessment of certainty of evidence using GRADE framework showed that overall level evidence evaluating effects of R18 administration in improving neurological outcome is moderate (Table 5).

**TABLE 3:** Subgroup analysis for R18 administration with respect to different stroke outcomes

Outcomes and subgroups	Number of analyses	Effect size		Heterogeneity (%) (*p*‐value)	Meta regression	
		SMD (95% CI)	*p*‐Value		Coef. (95% CI)	*p*‐Value
Neurological outcome						
Stroke model						
Permanent	12	0.67 (0.41–0.92)	<.0001	0.00 (.696)	Ref	—
Transient	1	0.64 (−0.39 to 1.69)	NA	NA	−0.02 (−1.06, 1.01)	.958
Interval between injury to treatment (min)						
0	4	0.80 (0.23–1.37)	.021	0.00 (.49)	Ref	—
30	2	0.38 (−7.94 to 8.71)	.661	69.70 (.06)	−0.39 (−1.19, 0.40)	.288
60	4	0.76 (0.35–1.16)	.010	0.00 (.87)	−0.04 (−0.69, 0.60)	.886
120	3	0.46 (0.07–0.85)	.035	0.00 (.90)	−0.33 (−1.01, 0.34)	.292
Follow‐up duration (hours)						
24	9	0.57 (0.25–0.88)	.003	0.00 (.755)	Ref	—
48	4	0.80 (0.23–1.37)	.021	0.00 (.491)	0.22 (−0.24, 0.70)	.314
Route of administration						
IP	4	0.80 (0.23–1.37)	.021	0.00 (.491)	Ref	—
IV and IA	9	0.57 (0.25–0.88)	.003	0.00 (.755)	−0.22 (−0.70, 0.24)	.314
Functional status						
Stroke model						
Permanent	10	0.61 (0.13–1.10)	.018	47.04 (.046)	Ref	—
Transient	7	0.45 (0.02–0.88)	.042	1.83 (.316)	−0.14 (−0.76, 0.47)	.623
Interval between injury to treatment (min)						
0	4	0.81 (0.31–1.30)	.014	0.00 (.611)	Ref	—
30	1	2.00 (0.86–3.13)	NA	NA	1.18 (0.09, 2.28)	.036
60	10	0.23 (−0.04 to 0.50)	.089	0.00 (.683)	−0.58 (−1.02, −0.14)	.013
120	2	1.19 (−2.23 to 4.63)	.141	0.00 (.481)	0.38 (−0.38, 1.15)	.301
Follow‐up duration (hours)						
24	7	0.59 (−0.22 to 1.41)	.127	64.14 (.010)	Ref	—
48	7	0.61 (0.26–0.96)	.005	0.00 (.518)	0.05 (−0.66, 0.77)	.870
120	3	0.37 (−0.70 to 1.44)	.276	7.47 (.343)	−0.19 (−1.10, 0.71)	.660
Route of administration						
IP	4	0.81 (0.31–1.30)	.014	0.00 (.611)	Ref	—
IV and IA	13	0.45 (0.06–0.83)	.024	38.12 (.060)	−0.37 (−1.02, 0.26)	.231
Infarct size						
Stroke model						
Permanent	16	−0.71 (−1.06, −0.35)	.001	38.13 (.045)	Ref	—
Transient	1	0.35 (−0.66 to 1.37)	NA	NA	1.06 (−0.43, 2.57)	.151
Interval between injury to treatment (min)						
0	4	−1.05 (−1.25, −0.86)	<.0001	0.00 (.965)	Ref	—
10	4	0.07 (−1.45 to 1.59)	.892	64.52 (.037)	1.12 (0.33, 1.91)	.009
30	2	−0.94 (−7.93 to 6.04)	.335	57.81 (.124)	0.18 (−0.72, 1.09)	.666
60	4	−1.08 (−1.34, −0.82)	.001	0.00 (.965)	−0.02 (−0.79, 0.73)	.942
120	3	−0.10 (−1.12 to 0.90)	.691	0.00 (.499)	0.94 (0.16, 1.73)	.022
Follow‐up duration (hours)						
4	4	0.07 (−1.45 to 1.59)	.892	64.52 (.037)	Ref	—
24	9	−0.69 (−1.14, −0.23)	.008	22.61 (.226)	−0.75 (−1.59, 0.08)	.074
48	4	−1.05 (−1.25, −0.86)	<.0001	0.00 (.965)	−1.12 (−2.02, −0.22)	.018
Route of administration						
IP	4	−1.05 (−1.25, −0.86)	<.0001	0.00 (.965)	Ref	—
IV and IA	13	−0.48 (−0.95, −0.17)	.043	50.12 (.020)	0.57 (−0.16, 1.31)	.119

Abbreviations: CI, confidence interval; Coef., coefficient; IA, intra‐arterial; IP, intraperitoneal; IV, intravenous; NA, not applicable; NR, not reported; Ref, reference category; SMD, standardized mean difference.

Regarding R18D, results of subgroup analysis demonstrated that R18D significantly improved neurological outcome only when administered 30 min after the injury (SMD = 0.76; 95% CI: 0.44–1.08; *p* = .002), whereas its administration immediately (SMD = 0.79; 95% CI: −0.25 to 1.84; *p* = .096), 60 min (SMD = −0.50; 95% CI: −7.19 to 6.17; *p* = .510) and 120 min (SMD = −0.15; 95% CI: −0.54 to 0.23; *p* = .126) after the injury did not result in significant resolvent in neurological outcome in animal models of stroke. The main sources of heterogeneity regarding the changes in neurological outcome following the administration of R18D were interval time between injury‐to‐treatment of 60 (Coef. = −1.25, 95% CI: −2.17 to −0.34, *p* = .012) and 120 (Coef. = −0.93, 95% CI: −1.83 to −0.02, *p* = .046) min (Table 4). Assessment of certainty of evidence using GRADE framework demonstrated that overall level evidence evaluating effects of R18D administration in improving neurological outcome is moderate (Table 5).

**TABLE 4:** Subgroup analysis for R18D administration with respect to different stroke outcomes

Subgroups	Number of analyses	Effect size		Heterogeneity (%) (*p*‐Value)	Meta regression	
		SMD (95% CI)	*p*‐Value		Coef. (95% CI)	*p*‐Value
Neurological outcome						
Interval between injury to treatment (min)						
0	4	0.79 (−0.25 to 1.84)	.096	60.39 (.055)	Ref	—
30	6	0.76 (0.44–1.08)	.002	0.00 (.738)	0.00 (−0.70, 0.70)	.999
60	2	−0.50 (−7.19 to 6.17)	.510	72.54 (.056)	−1.25 (−2.17, −0.34)	.012
120	2	−0.15 (−0.54 to 0.23)	.126	0.00 (.909)	−0.93 (−1.83, −0.02)	.046
Follow‐up duration (hours)						
24	1	1.26 (0.33–2.19)	NA	NA	Ref	—
48	13	0.40 (0.00–0.81)	.052	63.76 (.001)	−0.86 (−2.48, 0.76)	.270
Route of administration						
IP	13	0.40 (0.00–0.81)	.052	63.76 (.001)	Ref	—
IV	1	1.26 (0.33–2.19)	NA	NA	0.86 (−0.76, 2.48)	.270
Functional status						
Stroke model						
Permanent	14	0.41 (0.14–0.68)	.006	11.30 (.171)	Ref	—
Transient	6	0.19 (−0.01 to 0.40)	.062	0.00 (.951)	−0.21 (−0.64, 0.21)	.302
Interval between injury to treatment (min)						
0	4	0.45 (−0.02 to 0.93)	.057	0.00 (.637)	Ref	—
30	6	0.62 (0.00–1.23)	.048	34.77 (.114)	0.13 (−0.37, 0.65)	.582
60	8	0.25 (0.02–0.48)	.035	0.00 (.864)	−0.20 (−0.69, 0.28)	.390
120	2	−0.13 (−0.89 to 0.62)	.261	0.00 (.825)	−0.59 (−1.25, 0.06)	.076
Follow‐up duration (hours)						
24	1	2.03 (0.98–3.08)	NA	NA	Ref	—
48	16	0.33 (0.18–0.49)	<.0001	0.00 (.915)	−1.69 (−2.52, −0.86)	<.0001
120	3	0.08 (−0.50 to 0.67)	.596	0.00 (.723)	−1.94 (−2.84, −1.05)	<.0001
Route of administration						
IP	13	0.34 (0.14–0.53)	.002	0.00 (.775)	Ref	—
IV	7	0.39 (−0.20 to 0.99)	.156	46.86 (.066)	0.01 (−0.41, 0.44)	.933
Infarct size						
Interval between injury to treatment (min)						
0	4	−1.09 (−1.56, −0.62)	.005	0.00 (.694)	Ref	—
10	2	0.19 (−19.11 to 19.50)	.919	90.96 (.001)	1.23 (−0.25, 2.72)	.095
30	6	−1.11 (−1.46, −0.76)	<.0001	0.00 (.691)	−0.02 (−0.93, 0.89)	.962
60	2	−0.47 (−8.07 to 7.13)	.575	78.80 (.030)	0.63 (−0.55, 1.81)	.265
120	2	0.07 (−4.12 to 4.27)	.863	32.50 (.224)	1.17 (0.00, 2.35)	.050
Follow‐up duration (hours)						
4	2	0.19 (−19.11 to 19.50)	.919	90.96 (.001)	Ref	—
24	1	−1.31 (−2.25, −0.38)	NA	NA	−1.46 (−3.69, 0.75)	.178
48	13	−0.80 (−1.16, −0.43)	<.0001	54.22 (.01)	−0.95 (−2.43, 0.52)	.185
Route of administration						
IP	13	−0.80 (−1.16, −0.43)	<.0001	54.22 (.010)	Ref	—
IV	3	−0.33 (−4.66 to 3.99)	.771	88.52 (<.0001)	0.38 (−0.83, 1.59)	.513

Abbreviations: CI, confidence interval; Coef., coefficient; IA, intra‐arterial; IP, intraperitoneal; IV, intravenous; NA, not applicable; NR, not reported; Ref, reference category; SMD, standardized mean difference.

**TABLE 5:** Certainty of evidence with respect to R18 and R18D administration and the assessed outcomes

Type of outcome	Number of analyses	Risk of bias	Imprecision	Inconsistency (*I* ^2^ range)	Indirectness	Publication bias	Judgement	Level of evidence
R18								
Neurological outcome	17	Serious	No serious imprecision	No serious inconsistency*	No serious indirectness	No publication bias	Level of evidence was down rated one grade due to possible risk of bias.	Moderate
Functional status	13	Serious	No serious imprecision	No serious inconsistency*	No serious indirectness	No publication bias	Level of evidence was down rated one grade due to possible risk of bias.	Moderate
Infarct size	17	Serious	No serious imprecision	No serious inconsistency*	No serious indirectness	No publication bias	Level of evidence was down rated one grade due to possible risk of bias.	Moderate
R18D								
Neurological outcome	17	Serious	No serious imprecision	No serious inconsistency*	No serious indirectness	No publication bias	Level of evidence was down rated one grade due to possible risk of bias.	Moderate
Functional status	13	Serious	No serious imprecision	No serious inconsistency*	No serious indirectness	No publication bias	Level of evidence was down rated one grade due to possible risk of bias.	Moderate
Infarct size	17	Serious	No serious imprecision	No serious inconsistency*	No serious indirectness	No publication bias	Level of evidence was down rated one grade due to possible risk of bias.	Moderate

* There is no serious inconsistency since the sources of heterogeneity were identified.

### 3.5 Effects of polyarginine administration on functional status

**FIGURE 4:**
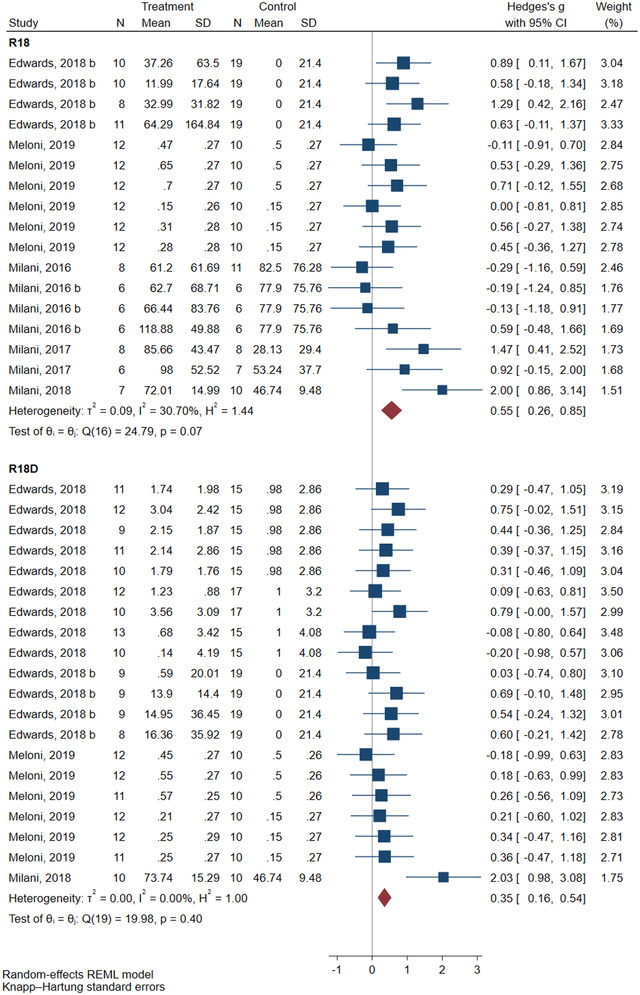
Efficacy of polyarginines’ administration on functional status changes following ischemic stroke. Results demonstrated that animals receiving R18 and R18D both had significantly improved functional status following ischemic stroke compared to that of control groups.

Figure 4 shows the meta‐analysis results regarding the improved functional status after administrating R18 and R18D. The results depicted a significant resolution of functional status in comparison with the control group following administration of R18 (SMD = 0.55; 95% CI: 0.26–0.85; *p* = .003) and R18D (SMD = 0.35; 95% CI: 0.16–0.54; *p* = .002) in animal models of stroke. No heterogeneity was observed regarding the findings of R18D (*I* ^2^ = 0.00%, *p* = .40). However, moderate heterogeneity was observed regarding the findings of R18 (*I* ^2^ = 30.70%, *p* = .07). The subgroup analysis for R18 and R18D is as follows.

Regarding R18, the results of the subgroup analysis depicted that the effect of R18 on functional status was at its highest level within 48 h after the injury (SMD = 0.61; 95% CI: 0.26–0.96; *p* = .005), compared to other follow‐up time intervals. Moreover, the efficacy was the highest when R18 was administered immediately after the injury (SMD = 0.81; 95% CI: 0.31–1.30; *p* = .014) compared to when it was administered 60 (SMD = 0.23; 95% CI: −0.04 to 0.50; *p* = .089) and 120 (SMD = 1.19; 95% CI: −2.23 to 4.63; *p* = .141) min after the injury, which were not effective. The main sources of heterogeneity regarding the changes in functional status following the administration of R18 were interval time between injury‐to‐treatment of 30 (Coef. = 1.18, 95% CI: 0.09–2.28, *p* = .036) and 60 (Coef. = −0.58, 95% CI: −1.02 to −0.14, *p* = .013) min (Table 3). Assessment of certainty of evidence using GRADE revealed a moderate level of evidence evaluating the effects of R18 in improving functional status in animal models of stroke (Table 5).

Regarding R18D, the results of the subgroup analysis revealed that R18D administration is efficient in functional status improvement in permanent ischemia (SMD = 0.41; 95% CI: 0.14–0.68, *p* = .006) rather than in transient ischemia (SMD = 0.19; 95% CI: −0.01 to 0.40; *p* = .062). Moreover, R18D was most effective when administered 30 (SMD = 0.62; 95% CI: 0.00–0.93; *p* = .048) and 60 (SMD = 0.25; 95% CI: 0.02–0.48; *p* = .035) min after the injury, in comparison with its administration immediately (SMD = 0.45; 95% CI: −0.02 to 0.93; *p* = .057) and 120 min (SMD = −0.13; 95% CI: −0.89 to 0.62; *p* = .261) after the injury. Furthermore, the resolvent of functional status was most apparent after 48 h of follow up (SMD = 0.33; 95% CI: 0.18–0.49; *p* < .0001) compared to 120 h (SMD = 0.08; 95% CI: −0.50 to 0.67; *p* = .596). Finally, the IP route of R18D administration was significantly more effective (SMD = 0.34; 95% CI: 0.14–0.53; *p* = .002) than its IV administration (SMD = 0.39; 95% CI: −0.20 to 0.99; *p* = .156) (Table 4). Certainty of evidence regarding the effects of R18D administration in improving functional status was moderate according to the GRADE framework (Table 5).

### 3.6 Effects of polyarginine administration on infarct size

**FIGURE 5:**
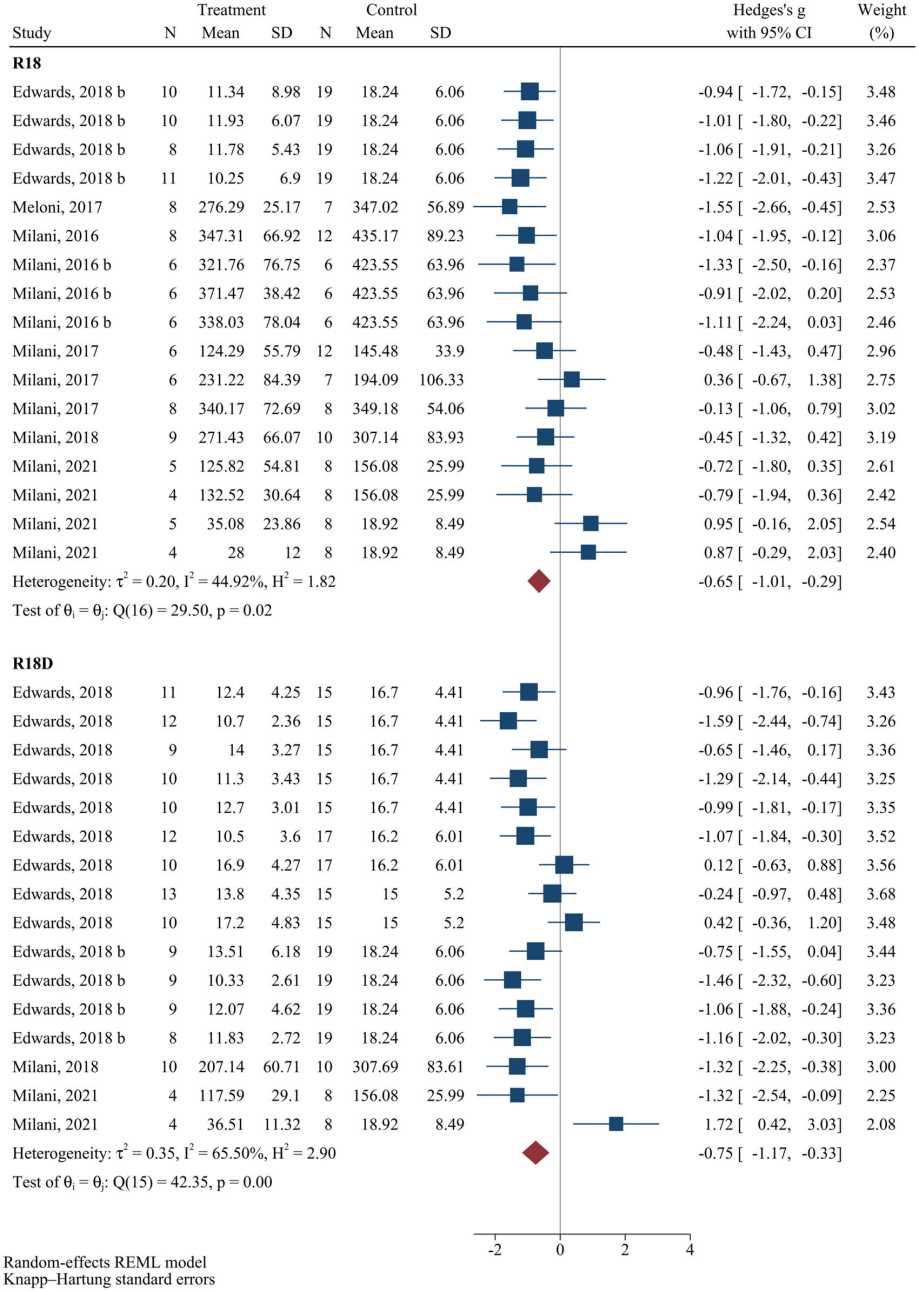
Efficacy of polyarginines’ administration on infarct size following ischemic stroke. Results demonstrated that R18 and R18D both significantly decreased infarct size following ischemic stroke.

Figure 5 shows the meta‐analysis results regarding the alternations in the infarct size after administrating R18 and R18D. The results showed that the infarct size was significantly decreased following administration of both R18 (SMD = −0.65; 95% CI: −1.01 to −0.29; *p* < .0001) and R18D (SMD = −0.75; 95% CI: −1.17 to −0.33; *p* < .0001) in animal models of stroke. Moderate heterogeneity was observed concerning the efficacy of R18 administration (*I* ^2^ = 44.92%, *p* = .02) and R18D administration (*I* ^2^ = 65.50%, *p* < .001) in decreasing infarct size in animal models of stroke. Results of subgroup analysis are as follows.

Regarding R18, results of subgroup analysis revealed that infarct size was significantly decreased when the polyarginine was administered immediately (SMD = −1.05; 95% CI: −1.25 to −0.86; *p* < .0001) and after 60 min (SMD = −1.08; 95% CI: −1.34 to −0.82; *p* = .001) after the injury, in contrary to when it was administered 10 (SMD = 0.07; 95% CI: −1.45 to −1.59; *p* = .892), 30 (SMD = −1.08; 95% CI: −7.93 to 6.04; *p* = .335), and 120 (SMD = −0.10; 95% CI: −1.12 to 0.90; *p* = .691) min after the injury. Moreover, the insignificant decrease in infarct size was only observed within 4 h after the injury (SMD = 0.07; 95% CI: −1.45 to 1.59; *p* = .892), in comparison with 24 h (SMD = −0.69; 95% CI: −1.14 to −0.23; *p* = .008) and 48 h (SMD = −1.05; 95% CI: −1.25 to −0.86; *p* < .0001), which were both significant. The main sources of heterogeneity regarding the decrease in infarct size following the administration of R18 were interval time between injury to treatment of 10 (Coef. = 1.12, 95% CI: 0.33–1.91, *p* = .009) and 120 (Coef. = 0.94, 95% CI: 0.16–1.73, *p* = .022) min and follow‐up time of 48 h (Coef. = −1.12, 95% CI: −2.02 to −0.22, *p* = .018) (Table 3). Determining the level of evidence evaluating the effects of R18 administration in decreasing infarct size in animal models of stroke using the GRADE framework revealed a moderate level of evidence (Table 5).

Regarding R18D, results of subgroup analysis demonstrated that infarct size was significantly decreased when the polyarginine was administered immediately (SMD = −1.09; 95% CI: −1.56 to −0.62; *p* = .005) and after 30 min (SMD = −1.11; 95% CI: −1.46 to −0.76; *p* < .0001) after the injury, in contrast to when it was administered 10 (SMD = 0.19; 95% CI: −19.11 to 19.50; *p* = .919), 60 (SMD = −0.47; 95% CI: −8.07 to 7.13; *p* = .575) and 120 (SMD = 0.07; 95% CI: −4.12 to 4.27; *p* = .863) min after the injury. Furthermore, the significant decrease was only observed 48 h after the injury (SMD = −0.80; 95% CI: −1.16 to −0.43; *p* < .0001), in comparison with that of 4 h after the injury (SMD = 0.19; 95% CI: −19.11 to 19.50; *p* = .919), which was not significant. Worth mentioning that data regarding the effects of R18D administration on infarct size were extracted from only one study, prohibiting us from performing a subgroup analysis regarding the specific subgroup. In addition, IP administration of R18D was significantly proficient in decreasing infarct size (SMD = −0.80; 95% CI: −1.16 to −0.43; *p* < .0001), in opposition to that of its IV administration (SMD = −0.33; 95% CI: −4.66 to 3.99; *p* = .771). In addition, the main source of heterogeneity concerning the R18D administration in decreasing infarct size was the 120 min time interval between injury and treatment (Coef. = 1.17, 95% CI: 0.00–2.35, *p* = .050) (Table 4). Determining the level of evidence evaluating the effects of R18D administration in decreasing infarct size in animal models of stroke using the GRADE framework revealed a moderate level of evidence (Table 5).

### 3.7 Effects of polyarginine administration on cerebral edema

**FIGURE 6:**
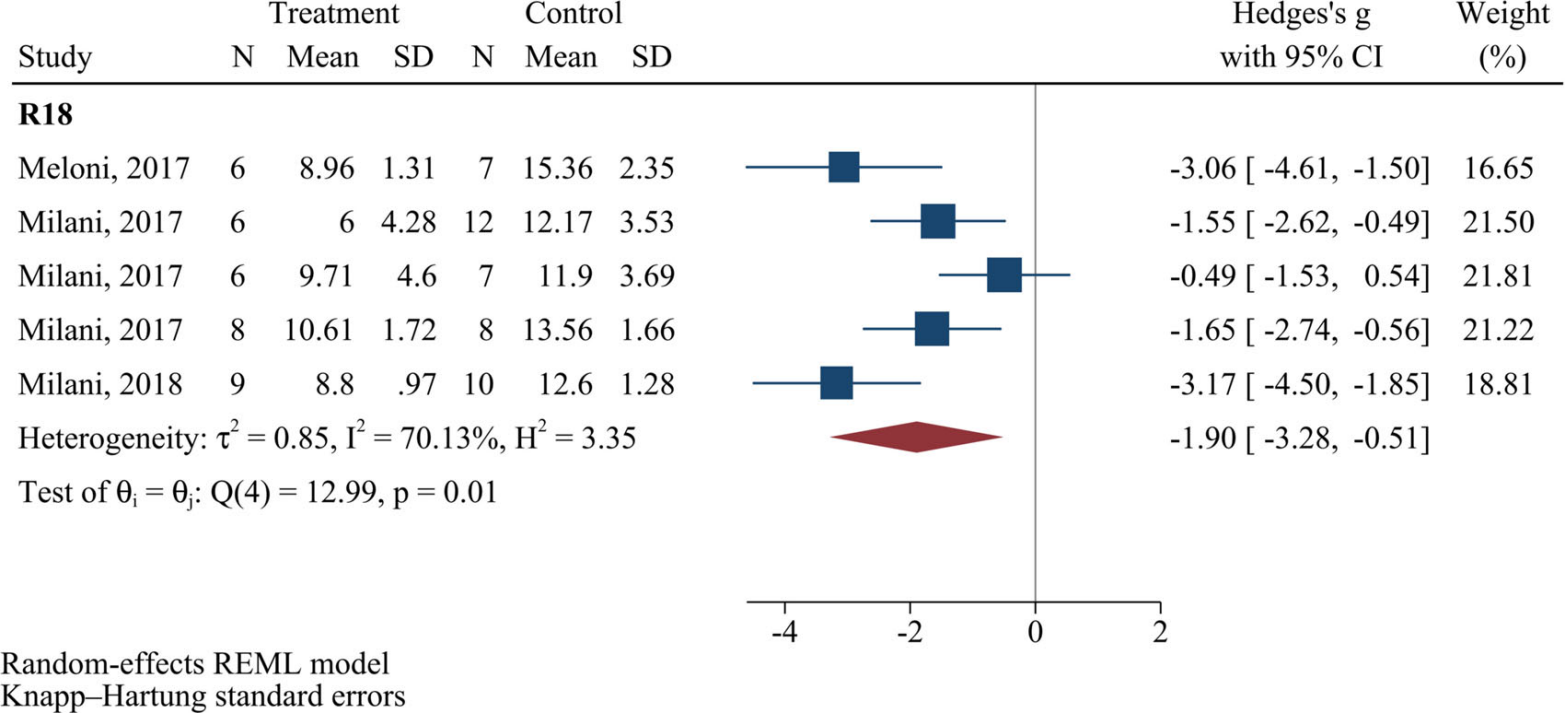
Efficacy of polyarginines’ administration on cerebral edema following ischemic stroke. Results demonstrated that R18 significantly improved cerebral edema following ischemic stroke.

Figure 6 shows the meta‐analysis results regarding the changes in the cerebral edema after administrating R18. A significant decrease was seen following the administration of R18 (SMD = −1.90; 95% CI: −3.28 to −0.51; *p* = .019). Furthermore, the reported results were observed with moderate heterogeneity (*I* ^2^ = 70.13%, *p* = .01).

## 4 DISCUSSION

This study aimed to systematically review the current animal studies conducted on the efficacy of polyarginine peptide administration in improving stroke outcomes. All the included studies were performed on rats. The results of our meta‐analysis showed a pattern of general improvement in stroke outcomes followed by the administration of specific subtypes of polyarginine peptides in animal models. In particular terms, brain edema infarct size, impaired functional status, and neurological outcomes in animal models of stroke were all improved, followed by administering certain polyarginines compared with the non‐treated control groups.

Although the precise mechanisms of CARPs‐induced neuro‐protection is yet be clarified, CARPs have been shown to trigger several neuroprotective mechanisms in stroke, including the ability to protect neurons from glutamate excitotoxicity and intracellular calcium influx (Meloni, Mastaglia, et al., 2020; MacDougall et al., 2019). They also reduce expression of calcium ion channels and their receptors (e.g., NMDA, AMPAR, TRPV1, NCX, CaV3.2, TNFR) in neuronal cells that may aggravate excitotoxicity following stroke (Meloni, Mastaglia, et al., 2020). Other neuroprotective actions of arginine‐rich peptides seem to be due to their effects on mitochondria. Previous studies revealed that CARPs reduce complex I activity and production of reactive oxygen species, leading to increased tolerance to calcium influx and the opening of the mitochondrial permeability transition pore, increasing ATP recovery and reducing cytochrome c release (Horton et al., 2008). CARPs may also play their neuroprotective effects through inhibition of proteolytic enzymes such as cathepsin C, furin, and the proteasome, and by binding to the apelin receptor to induce pro‐survival cell signaling pathways (Edwards et al., 2018b).

As one of the most prevalently administered polyarginines, R18 significantly improved all four evaluated outcomes in animal stroke models. In specific terms, R18 significantly decreased infarct size in animal stroke models. These results may be due to its neuroprotective and anti‐inflammatory effects, previously described in in vitro studies (Chiu et al., 2019; Meloni, Brookes, et al., 2015). Consequently, this decrease in infarct size may be responsible for improving neurological outcome and functional status, observed in our findings concerning rats. In addition, CARPs effects on neurological improvement were not subjected to heterogeneity, and a moderate level of evidence supported its efficacy in improving neurological outcomes and decreasing infarct size. Astonishingly, contemplating on the results of other articles studying different animal models of stroke, Meloni et al. observed significant improvement concerning the administration of CARPs in monkeys’ model of stroke in terms of both infarct size and functional status. However, since only one study was conducted on these primates, and all other studies were on rats, we omitted their selection to the present study for the purpose of keeping the final results more homogenous (Meloni, Chen, et al., 2020). Nevertheless, more studies on primates in this topic are recommended, since their CNS resembles the most to that of the humans, and a statistical conclusion on whether CARPs could resolve at least part of the injury in brain following stroke is well deserved.

Interestingly, these beneficial effects of R18 on the evaluated outcomes were not observed within the first 4 h after the ischemia. This may be attributed to its ongoing neuroprotection effect, which can present in the long term. Moreover, the beneficial effects of R18 on stroke outcomes were mainly observed, while the CARP was administered immediately after the injury. These findings may be due to the inhibitory effects of polyarginines in the initiation of neuroinflammation in the injury area, which was demonstrated in previous studies (Chiu et al., 2019). However, the existing evidence regarding the matter was subjected to certain limitations. First, the number of studies inducing transient ischemia in animal models was considerably low. Thus, future studies may adopt different strategies to induce stroke in animal models to shed light on the neuroprotective effects of R18. Second, the number of studies evaluating its effects on decreasing cerebral edema was also considerably low, making it a possible candidate for assessment in future studies. Finally, a moderate level of evidence supported the beneficial effects of R18 administration on improving functional status following stroke induction due to possible risk of bias. Thus, future studies are recommended to fill the mentioned gaps in the existing evidence.

Regarding the therapeutic effects of R18D, an enantiomer of R18, the results showed that this polyarginine could also effectively improve stroke outcomes. Specifically, R18D significantly decreased infarct size. Furthermore, the analysis results on the reviewed studies demonstrated a significant resolvent in neurological outcomes and functional status following administration of R18D, which could be a consequence of the reduction in the infarct size. However, some studies have proposed mechanisms, other than decreasing the infarct zone, involved with the resolvent observed followed by R18D administration in animal stroke models (Edwards et al., 2018a). In addition to evidence regarding R18, studies on R18D were subjected to limitations. First, the studies did not evaluate the effects of R18D administration on the changes in the infarct size and functional outcomes following different models of stroke (transient or permanent). Second, brain edema was not evaluated following R18D administration. As for a comparison between R18 and R18D, the improvements observed following R18 administration were more significant. Both R18 and R18D are hypothesized to exert beneficial effects by reducing excitotoxicity through endocytic internalization of neural cells’ surface ion channels (Meloni, Milani, et al., 2015).

Despite adding novel evidence to the current literature, the result of this meta‐analysis should be carefully generalized because of some of its limitations. The first and most crucial limitation is that the results of animal experiments are not reliable to be generalized to patient management due to several factors, including different pathophysiology and involved metabolic pathways of the underlying condition. Second, animal studies may have a low quality of study design in various aspects, including model formation and a restricted number of experiments. Third, due to restricted follow‐up duration, the long‐time effects of administrating polyarginine peptides remain unclear. Future studies are warranted to address the long‐term behavioral and neurological improvements. In addition, it was not possible to draw a conventional pattern of dose‐response based on the current evidence. Therefore, it would be purposeful to utilize a wider dose range of the peptide in the future.

Furthermore, the models applied across the studies varied in some cases; for instance, two of the included studies used the Rice‐Vannucci HIE model while others did not. Moreover, the means of drug administration was IV in the majority of the studies, and IP and IC administration were not adequately used in the included studies. Worth mentioning that one study did apply intra‐arterial route of transplantation in one of its experiment sets, which again could be the source of disparity, as opposed to other studies using the tail vein. Altogether, these disparities among the studies can encourage and inspire further research projects, with the aim of unifying their methods of evaluations and treatment.

## 5 CONCLUSION

The present study results showed promising effects regarding the efficacy of administering polyarginines of R18 and R18D in improving functional status and neurological outcomes and decreasing infarct size following the induction of stroke in rats. Moreover, R18 effectively reduced brain edema in animal models of stroke. All of the mentioned effects were supported by a moderate level of evidence. However, while these results are subjected to certain limitations, the pre‐clinical data are highly encouraging and provide the support for the translation of polyarginine peptides as possible clinical stroke therapeutics.

## AUTHOR CONTRIBUTIONS

*Study design*: Mahmoud Yousefifard and Yaser Azizi. *Data gathering*: Fateme Tahmasbi, Mahta Mardani, Arian Madani Neishabouri, Amirmohammad Toloui, and Mahmoud Yousefifard. *Analysis*: Mahmoud Yousefifard and Mahta Mardani. *Interpreting the results*: All authors. *Drafting of the manuscript*: Fateme Tahmasbi, Arian Madani Neishabouri, and Mahta Mardani. *Critical revision*: Mahmoud Yousefifard, Yaser Azizi, and Amirmohammad Toloui.

## CONFLICTS OF INTEREST

The authors declare no conflict of interest.

### PEER REVIEW

The peer review history for this article is available at https://publons.com/publon/10.1002/brb3.2858

## Supporting information

Appendix 1: Search queries for databases

## Data Availability

All data generated or analyzed during this study are included in the article.
