# Supplementary material for: Efficacy of polyarginine peptides in the treatment of stroke: A systematic review and meta‐analysis
Source: Brain Behav. 2022 Dec 21;13(1):e2858. doi: 10.1002/brb3.2858 (PMC9847609; doi:10.1002/brb3.2858)
Supplement: Supplementary file 1 — Appendix 1: Search queries for databases [file BRB3-13-e2858-s001.docx]

**Appendix 1: Search queries for databases**

1. **Embase**

('cerebrovascular accident'/exp OR 'cva' OR 'accident, cerebrovascular' OR 'acute cerebrovascular lesion' OR 'acute focal cerebral vasculopathy' OR 'acute stroke' OR 'apoplectic stroke' OR 'apoplexia' OR 'apoplexy' OR 'blood flow disturbance, brain' OR 'brain accident' OR 'brain attack' OR 'brain blood flow disturbance' OR 'brain insult' OR 'brain insultus' OR 'brain vascular accident' OR 'cerebral apoplexia' OR 'cerebral insult' OR 'cerebral stroke' OR 'cerebral vascular accident' OR 'cerebral vascular insufficiency' OR 'cerebro vascular accident' OR 'cerebrovascular accident' OR 'cerebrovascular arrest' OR 'cerebrovascular failure' OR 'cerebrovascular injury' OR 'cerebrovascular insufficiency' OR 'cerebrovascular insult' OR 'cerebrum vascular accident' OR 'cryptogenic stroke' OR 'ischaemic seizure' OR 'ischemic seizure' OR 'stroke' OR 'thrombotic stroke' OR 'stroke patient'/exp OR 'stroke patient' OR 'stroke sufferer' OR 'stroke sufferers' OR 'ischemic stroke'/exp OR 'ischaemic stroke' OR 'ischemic stroke' OR 'brain infarction'/exp OR 'brain infarct' OR 'brain infarction' OR 'cerebral infarct' OR 'cerebral infarction' OR 'cerebrovascular infarction' OR 'cortical infarction' OR 'hemisphere infarct' OR 'hemisphere infarction' OR 'hemispheric infarct' OR 'hemispheric infarction' OR 'infarction, brain' OR 'silent brain infarction') AND ('arginine-rich peptide or' OR 'arginine-rich protein' OR 'polyarginine'/exp OR 'poly (arginine)' OR 'poly (l arginine)' OR 'poly 1 arginine' OR 'poly arginine' OR 'poly l arginine' OR 'poly levo arginine' OR 'polyarginine' OR 'polyarginine sulfate' OR 'polyarginine sulphate' OR 'polylysine'/exp OR 'isopolylysine' OR 'lysine polymer' OR 'poly (l lysine)' OR 'poly (lysine)' OR 'poly dl lysine' OR 'poly l lysine' OR 'poly l lysine hydrobromide' OR 'poly levo lysine' OR 'poly lysine' OR 'polylysin' OR 'polylysine' OR 'polylysine hydrobromide' OR 'cationic arginine-rich peptide' OR 'arginine-rich cell-penetrating peptide' OR 'neuroprotective peptide')

1. **PubMed**

("Stroke"[mh] OR "Brain Infarction"[mh] OR "Stroke Rehabilitation"[mh] OR "Brain Stem Infarctions"[mh] OR "Infarction, Anterior Cerebral Artery"[mh] OR "Cerebral Infarction"[mh] OR "Hypoxia-Ischemia, Brain"[mh] OR "Brain Ischemia"[mh] OR Stroke[tiab] OR Brain Infarction[tiab] OR Stroke Rehabilitation[tiab] OR Brain Stem Infarctions[tiab] OR Infarction, Anterior Cerebral Artery[tiab] OR Cerebral Infarction[tiab] OR Hypoxia-Ischemia, Brain[tiab] OR Brain Ischemia[tiab] OR Cerebrovascular Accident[tiab] OR Brain Vascular Accident[tiab] OR Cerebrovascular Stroke[tiab]) AND ((polyarginine[Title/Abstract] OR poly-arginine[Title/Abstract] OR "poly-arginine-9"[Title/Abstract] OR "arginine-rich"[Title/Abstract] OR "arginine-rich peptide"[Title/Abstract] OR "arginine-rich protein"[Title/Abstract] OR "cationic arginine-rich peptide"[Title/Abstract] OR "arginine-rich CPP"[Title/Abstract] OR "arginine-rich cell-penetrating peptide"[Title/Abstract] OR "neuroprotective peptide"[Title/Abstract] OR "neuroprotective protein"[Title/Abstract]))

1. **Scopus**

(TITLE-ABS-KEY ( polyarginine OR "poly-arginine" OR "poly-arginine-9" OR "arginine-rich" OR "arginine-rich peptide" OR "arginine-rich protein" OR "cationic arginine-rich peptide" OR "arginine-rich CPP" OR "arginine-rich cell-penetrating peptide" OR "neuroprotective peptide" OR "neuroprotective protein" ) AND TITLE-ABS-KEY ( stroke OR "Brain Infarction" OR "Stroke Rehabilitation" OR "cryptogenic stroke" OR "Brain Stem Infarctions" OR "Anterior Cerebral Artery Infarction" OR "Posterior Cerebral Artery Infarction" OR "Cerebral Infarction" OR "cerebrovascular failure" OR "cerebrovascular injury" OR "cerebrovascular insufficiency" OR "cerebrovascular insult" OR "cerebrum vascular accident" OR "Brain Hypoxia-schemia" OR "Brain Ischemia" OR "Brain Infarction" OR "Cerebrovascular Accident" OR "cerebro vascular accident" OR "cerebrovascular arrest" OR "Brain Vascular Accident" OR "Cerebrovascular Stroke" OR "Acute Stroke" OR "acute focal cerebral vasculopathy" OR "apoplectic stroke" OR "Brain Attack" OR "apoplexia" OR "cerebral apoplexia" ) )

1. **Web of Science**

(TOPIC: (TOPIC: ((((((((polyarginine OR poly-arginine) OR poly-arginine-9) OR arginine-rich) OR arginine-rich peptide) OR arginine-rich protein) OR arginine-rich cell-penetrating peptide) OR "neuroprotective peptide") OR "neuroprotective protein") AND TOPIC: ((((((((((((((((((((((((((stroke OR "Brain Infarction") OR "Stroke Rehabilitation") OR "cryptogenic stroke") OR "Brain Stem Infarctions") OR "Anterior Cerebral Artery Infarction") OR "Posterior Cerebral Artery Infarction") OR "Cerebral Infarction") OR "cerebrovascular failure") OR "cerebrovascular injury") OR "cerebrovascular insufficiency") OR "cerebrovascular insult") OR "cerebrum vascular accident") OR "Brain Hypoxia-schemia") OR "Brain Ischemia") OR "Brain Infarction") OR "Cerebrovascular Accident") OR "cerebro vascular accident") OR "cerebrovascular arrest") OR "Brain Vascular Accident") OR "Cerebrovascular Stroke") OR "Acute Stroke") OR "acute focal cerebral vasculopathy") OR "apoplectic stroke") OR "Brain Attack") OR "apothecia") OR "cerebral apothecia"))

Timespan: All years. Databases: WOS, KJD, RSCI, SCIELO.
